# Patient’s and physician’s awareness of kidney disease in coronary heart disease patients – a cross-sectional analysis of the German subset of the EUROASPIRE IV survey

**DOI:** 10.1186/s12882-017-0730-3

**Published:** 2017-10-25

**Authors:** Martin Wagner, Christoph Wanner, Martin Schich, Kornelia Kotseva, David Wood, Katrin Hartmann, Georg Fette, Viktoria Rücker, Mehmet Oezkur, Stefan Störk, Peter U. Heuschmann

**Affiliations:** 1 Institute of Clinical Epidemiology and Biometry, University of Würzburg, Petrinistr. 33a, 97080 Würzburg, Germany; 2 Division of Nephrology, Department of Medicine I, University Hospital Würzburg, Würzburg, Germany; 3 Comprehensive Heart Failure Center, University of Würzburg, Würzburg, Germany; 4 Department of Cardiovascular Medicine, National Heart and Lung Institute, Imperial College London, London, UK; 5 Department of Public Health, University of Ghent, Ghent, Belgium; 6 Institute of Informatics VI, University of Würzburg, Würzburg, Germany; 7 Department of Cardiovascular Surgery, University Hospital Würzburg, Würzburg, Germany; 8 Division of Cardiology, Department of Medicine I, University Hospital Würzburg, Würzburg, Germany; 9 Clinical Trial Center, University Hospital Würzburg, Würzburg, Germany; 10 Fellow of the European Society of Cardiology, Sophia Antipolis, France

**Keywords:** Coronary heart disease, Chronic kidney disease, Patients’ awareness, Physicians’ awareness, ICD-coding of CKD, EUROASPIRE survey

## Abstract

**Background:**

Chronic kidney disease (CKD) is a common comorbid condition in coronary heart disease (CHD). CKD predisposes the patient to acute kidney injury (AKI) during hospitalization. Data on awareness of kidney dysfunction among CHD patients and their treating physicians are lacking. In the current cross-sectional analysis of the German EUROASPIRE IV sample we aimed to investigate the physician’s awareness of kidney disease of patients hospitalized for CHD and also the patient’s awareness of CKD in a study visit following hospital discharge.

**Methods:**

All serum creatinine (SCr) values measured during the hospital stay were used to describe impaired kidney function (eGFR_CKD-EPI_ < 60 ml/min/1.73m^2^) at admission, discharge and episodes of AKI (KDIGO definition). Information extracted from hospital discharge letters and correct ICD coding for kidney disease was studied as a surrogate of physician’s awareness of kidney disease. All patients were interrogated 0.5 to 3 years after hospital discharge, whether they had ever been told about kidney disease by a physician.

**Results:**

Of the 536 patients, 32% had evidence for acute or chronic kidney disease during the index hospital stay. Either condition was mentioned in the discharge letter in 22%, and 72% were correctly coded according to ICD-10. At the study visit in the outpatient setting 35% had impaired kidney function. Of 158 patients with kidney disease, 54 (34%) were aware of CKD. Determinants of patient’s awareness were severity of CKD (OR_eGFR_ 0.94; 95%CI 0.92–0.96), obesity (OR 1.97; 1.07–3.64), history of heart failure (OR 1.99; 1.00–3.97), and mentioning of kidney disease in the index event’s hospital discharge letter (OR 5.51; 2.35–12.9).

**Conclusions:**

Although CKD is frequent in CHD, only one third of patients is aware of this condition. Patient’s awareness was associated with kidney disease being mentioned in the hospital discharge letter. Future studies should examine how raising physician’s awareness for kidney dysfunction may improve patient’s awareness of CKD.

## Background

Chronic kidney disease (CKD) has been identified as a common and important risk factor in patients with coronary heart disease (CHD) [1–4]. Patients with CKD represent a multi-morbid population [5] which is at risk for various complications, e.g. episodes of acute kidney injury (AKI), in hospital stays of various causes, including CHD [6]. CKD and AKI impact independently on morbidity and mortality, even if classic cardiovascular risk factors, such as hypertension, diabetes, and dyslipidemia are controlled [7–10]. The health economic relevance of kidney disease, acknowledging multi-morbidity of patients and risk for complications, is also reflected by the fact that adequate ICD-10 coding for CKD and AKI impacts on reimbursement [11].

Early referral to nephrology or specialist care for impaired kidney function is associated with a reduced risk of CHD events, CKD progression, and mortality [12]. To decide on treatment goals and educate the patient, nephrology care suggests determining the risk of an individual patient for disease progression [13]. The awareness of CKD in the general population and in CKD cohorts is limited [14, 15]. It is suggested that well-informed patients aware of their disease may show better adherence to medication and may easier achieve treatment targets [16, 17].

In CHD, many patients and physicians know about of the importance of classic cardiovascular (CV) risk factors, treatment targets, and lifestyle advice such as smoking cessation, diet, or physical activity [18, 19]. However, implementation of guideline recommendations into daily practice is still far from optimal [20]. Evidence is sparse regarding the perception of kidney disease in patients with CHD [21], both from the perspective of patients as of physicians. Herein, to report on important inhospital events including e.g. chronic and/or acute deterioration of kidney function in discharge letters is important to transfer information from the hospital to the ambulatory setting.

In the current study, we analyzed (a) how chronic and/or acutely impaired kidney function is reported in the discharge letter after hospitalization for CHD and (b) the completeness of ICD coding for CKD and AKI as reflection the physician’s awareness of kidney disease. We also describe the level of CKD awareness in CHD patients in the ambulatory setting following hospital discharge.

## Methods

### Patient population and study setting

We used data of the German sample (*n* = 536) of the EUROASPIRE IV survey [20]. The EUROpean Action on Secondary and Primary Prevention by Intervention to Reduce Events surveys are a multinational initiative of the European Society of Cardiology and the European Association for Cardiovascular Prevention and Rehabilitation to assess quality of secondary prevention in CHD patients across Europe [19]. The study design of the EUROASPIRE IV “hospital-arm” has been reported previously [20]. Briefly, for each participating country, a geographical region with > 0.5 million people was selected in which at least one hospital offering interventional cardiology and cardiac surgery and one or more acute hospitals admitting patients with MI and myocardial ischemia. All patients hospitalized for acute or elective treatment of CHD (coronary artery bypass grafting [CABG], percutaneous coronary intervention [PCI], acute myocardial infarction, or myocardial ischemia) were identified from the hospital’s patient management systems and invited to participate in the study. This “index” CHD-event could represent the primary diagnosis of CHD as well as any subsequent episode in previously established CHD. Subjects were eligible if they were 18–79 years old and the study visit took place between 6 and 36 months after the index hospital stay. All participants provided written informed consent. At the German study center, patients were recruited from the University Hospital Wuerzburg (Dept. of Medicine I and Department for Thoracic and Cardiovascular Surgery) and the Klinik Kitzinger Land (Dept. of Medicine). The study protocol and data-handling at the German study center were approved by the Ethics Committee of the Medical Faculty of the University of Würzburg (Vote 58/12) and the data protection officers of the University Hospital and the University of Würzburg (DS-117.605-15/12).

### Data collection

Information on the CHD event, risk factors, clinical measurements and laboratory values (e.g. SCr at hospital admission) were obtained by retrospective review of index hospitalization charts. During the study visit, details of CHD history (e.g. MI, CABG, PCI/stent) and information on co-morbid conditions, medication, life-style and behavior were collected in personal interviews, and standardized examinations were performed according to the EUROASPIRE IV protocol, including blood pressure, weight, height, carbon monoxide (CO) in exhaled air [22]. Serum creatinine (SCr), lipid profile and HbA1c were analyzed centrally from fasting blood samples at the National Public Health Institute, Helsinki, Finland. In addition, urinary albumin/creatinine ratio (ACR) was measured locally.

In addition to the core EUROASPIRE IV protocol, we implemented a *kidney module* at the German study center a few weeks after the start of enrollment, thus respective information was missing in *n* = 62 (11.6%) individuals. Additional information relating to kidney function during the index hospitalization, i.e. details of CKD or AKI, including dialysis requirement, reported in the discharge letter, was collected by chart review. For all German patients we were able to retrospectively collect laboratory data on SCr at hospital discharge and the maximum value of SCr during the hospital stay. For patients admitted to the University Hospital Würzburg (*n* = 498) the Data Warehouse of the Comprehensive Heart Failure Center [23] was utilized, e.g. for extraction of SCr values, ICD-10 codes for CKD and AKI, and OPS-codes for dialysis treatment. At the study visit, we collected data on the patient’s awareness of CKD and specialist care during personal interviews. It included the following questions: “Have you ever been told by a doctor/health care provider that your kidney function is impaired, e.g. not as good as it would be expected?”; “Have you ever been told by a doctor/health care provider that you should be seen by a specialist to have your kidney function checked?”; “Have you ever been seen by a specialist to have your kidney function checked and/or treated?”.

### Presence of CKD

**Fig. 1:**
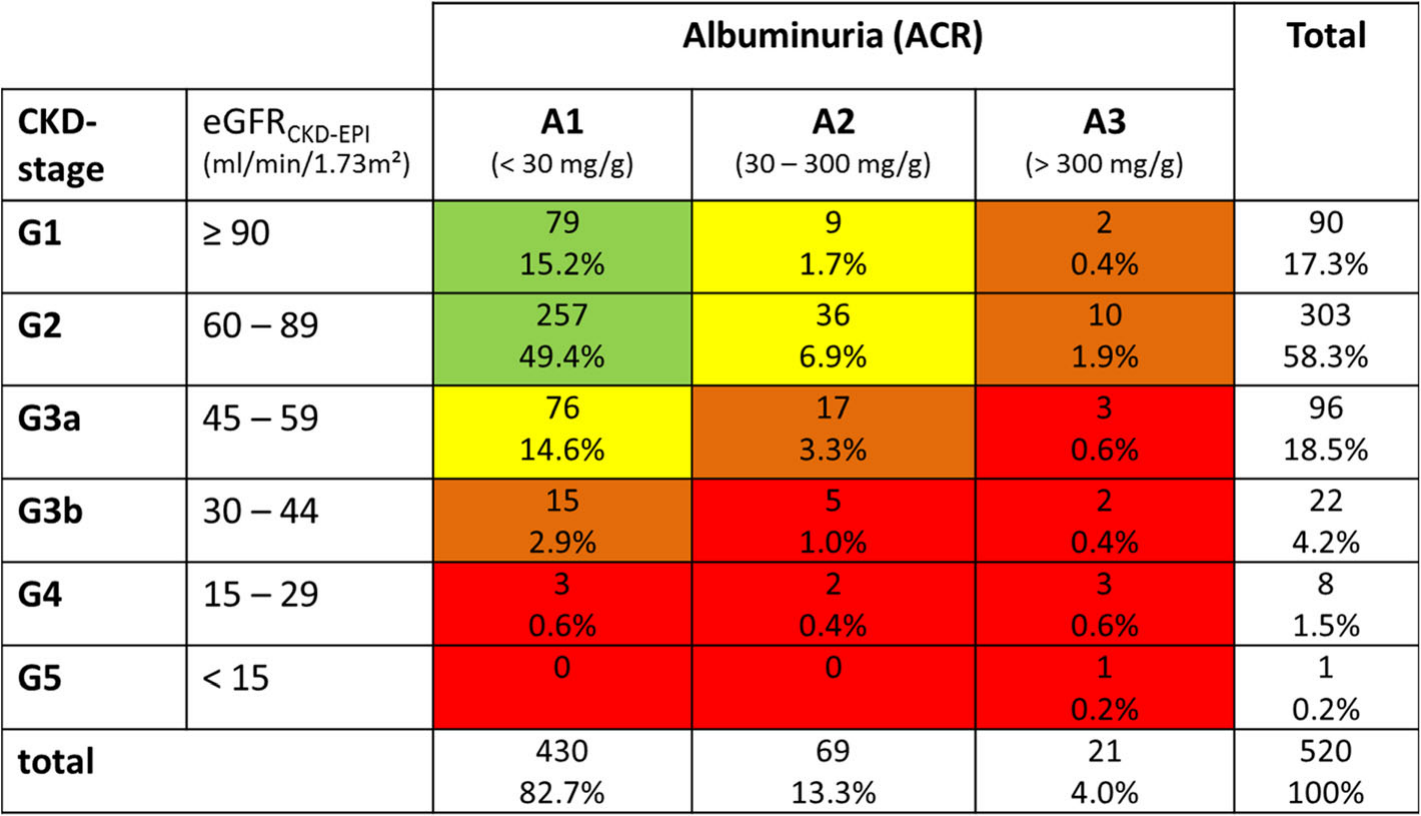
Stages of CKD according to eGFR and albuminuria following KDIGO classification; displayed are number of patients (%) within each category. The color code indicates risk category according to KDIGO [24]: green “low risk”, yellow “moderate risk”, orange “high risk” and red “very high risk”

Kidney function was categorized into CKD-G and CKD-A stages based on estimated glomerular filtration rate (eGFR_CKD-EPI_) and ACR according to KDIGO (Kidney Disease: Improving Global Outcomes) [24] (Fig. 1). Due to 95.5% missing data on ACR in the hospital records, impaired kidney function during the hospital stay was described as eGFR_CKD-EPI_ < 60 ml/min/1.73m^2^ at hospital admission or at discharge or any episode of AKI. AKI during the index hospital stay was defined as SCr increase of ≥ 0.3 mg/dl within 48 h or SCr increase of 1.5–1.99× baseline SCr within 7 days (KDIGO AKI stage 1), SCr-increase of 2.0–2.9× baseline SCr (stage 2) and SCr-increase ≥ 3.0× baseline or SCr > 4 mg/dl or dialysis (stage 3) [25]. A binary variable CKD at the study visit was defined as all CKD-G stages G3a and higher and CKD stages G1A3, G2A2, G2A3 (i.e., largely preserved GFR but significant albuminuria).

### Outcome (awareness)

The fact that CKD or AKI was explicitly stated in prominent parts in the discharge letter (diagnoses and/or summary) and the completeness of ICD coding (i.e., correct coding of CKD [ICD-10 codes N18, N19, I12.0, I13) and/or AKI [ICD-10 code N17]) was used to operationalize *physician’s awareness of kidney disease.*

*Patients’ awareness of CKD* was defined as positive response to the first question of the kidney module: “Have you ever been told by a doctor/health care provider that your kidney function is impaired, e.g. not as good as it would be expected?”.

### Covariates

During the index hospital stay the following risk factors were defined: history of heart failure (according to case history or echocardiographic findings of cardiac dysfunction at admission); cardiovascular risk factors known at admission or reported in the discharge letter as hypertension, dyslipidemia, diabetes, smoking and obesity (BMI ≥ 30 kg/m^2^ at admission or explicitly stated in the discharge letter). Definitions of risk factors at the study visit were as follows: hypertension (blood pressure ≥ 140/90 mmHg, ≥ 140/85 mmHg in diabetes, ≥ 150/90 mmHg in patients > 80 yrs., ≥130/90 mmHg in patients with CKD [26]), diabetes (self-reported diabetes or impaired fasting glucose or impaired glucose tolerance), dyslipidemia (LDL cholesterol ≥2.5 mmol/L), obesity (body mass index [BMI] ≥30 kg/m^2^) and smoking (self-reported, or CO in exhaled air >10 ppm [20]).

### Statistical methods

Data are reported as proportions and median (inter-quartile range, IQR) and were compared across categories of interest (i.e. impaired kidney function during hospital stay, CKD at study visit) using Wilcoxon rank-sum test, Kruskal-Wallis test and χ^2^ test/Fisher’s exact test, as appropriate. The trend of patient’s awareness, referral to specialist and visit at the specialist across CKD-G stages was analyzed by Cochran-Mantel-Haenszel test. Determinants of *patients’ awareness of CKD* (i.e. positive response to the first question of the kidney module: “Have you ever been told by a doctor/health care provider that your kidney function is impaired, e.g. not as good as it would be expected?”) and the fact that CKD or AKI were mentioned in the hospital discharge letter (*physician’s awareness of kidney disease)* in all participants were investigated by univariable and multivariable logistic regression, using backwards selection including variables statistically significant in univariate analysis. Results are displayed as odds ratio (OR) with respective 95% confidence intervals (CI). Analyses were performed on a complete case dataset, i.e. *n* = 474 with data on the *kidney module*. We tested the robustness of the multivariable models on a dataset with imputed missing data, using five imputations derived from the Markov Chain Monte Carlo method (SAS proc. mi). We also performed sensitivity analyses in which eGFR <50 ml/min/1.73 m^2^ was used rather than <60 ml/min/1.73m^2^ to increase the confidence in the patient suffering from true kidney disease by excluding patients with only a minor variation in SCr that made their eGFR values being slightly below 60 ml/min/1.73m^2^. Analyses were performed using SAS 9.3 (SAS Institute Inc., Cary, NC, USA). Two-sided *p*-values <0.05 were considered statistically significant.

## Results

### Prevalence of impaired kidney function during the (index) hospital stay

A total of 536 German patients were enrolled in EUROASPIRE IV (median age at the index hospital stay 67 years, 82% male). Median SCr at hospital admission for the index CHD event (i.e. first measurement in patient record) was 0.9 (IQR 0.8; 1.1) mg/dl, reflecting an eGFR_admission_ of 81.1 (66.2; 93.3) ml/min/1.73m^2^ and 94 patients (17.6%) had eGFR <60 ml/min/1.73 m^2^. At hospital discharge (i.e. last measurement in patient record), median SCr was 1.0 (0.8; 1.1) mg/dl, median eGFR_discharge_ was 78.7 (64.1; 91.4) ml/min and 100 (18.1%) patients had eGFR <60 ml/min/1.73m^2^. AKI was observed in 94 (18.1%) subjects. Most AKI episodes represented a slight increase of SCr (AKI stage 1: 89.4%), while a rise in SCr of not more than exactly 0.3 mg/dl was observed in 16 patients. Two events were of AKI stage 2 (2.1%) and 8 (8.5%) were of stage 3, of which 3 had to be treated by acute hemodialysis. Any impairment in kidney function during the hospital stay (either eGFR <60 ml/min/1.73m^2^ at admission, or at discharge or AKI) was observed in 172 patients (32.2%). Patients with impaired kidney function were older, more often had a history of heart failure, had a longer duration of CHD, and were more likely to receive CABG during the hospital stay. The hospital stay was on average also longer in CKD patients as compared to patients without renal impairment (Table 1). No differences in classic CV risk factors were observed. In the sensitivity analysis applying the lower eGFR cut-off, we found 38 (7.1%) subjects with eGFR <50 ml/min/1.73m^2^ at hospital admission, *n* = 46 (8.7%) at hospital discharge and any impairment of kidney function during the hospital stay in *n* = 119 (22.3%) patients. We did not find any meaningful differences in the patient characteristics as compared to results using the eGFR 60 ml/min/1.73m^2^ cut-off (detailed data not shown).

**Table 1:** Patient’s characteristics during the EUROASPIRE IV index hospital stay by impaired kidney function

	Normal kidney function *n* = 362 (67.8%)	Impaired kidney function *n* = 172 (32.2%)	*p*-value
Age, years	64.0 (58.5; 70.4)	72.0 (65.7; 75.1)	<0.001
Male sex	301 (83.2%)	139 (80.8%)	0.51
Length of hospital stay (days)	3 (1; 7)	8 (3; 11)	<0.001
Details of CHD event during index hospital stay			
AMI, any	128 (35.5%)	75 (43.6%)	0.07
NSTEMI	53 (41.4%)	33 (44.0%)	0.26
STEMI	60 (46.9%)	28 (37.3%)	
Unclear/missing	15 (11.7%)	14 (18.7%)	
Therapy (max.)			<0.001
Conservative (no intervention)	54 (14.9%)	29 (16.9%)	
PCI/stent	279 (77.1%)	88 (51.2%)	
CABG	29 (8.0%)	55 (32.0%)	
CHD history			
Index event as the primary diagnosis of CHD	162 (44.8%)	64 (37.2%)	0.10
In those with h/o CHD			
Duration of CHD, yrs.	1.4 (0.4; 9.0)	5.9 (0.6; 16.3)	<0.01
CABG (prior to index)	35 (17.5%)	21 (19.4%)	0.67
PCI/stent (prior to index)	101 (50.5%)	55 (50.9%)	0.94
MI (prior to index)	125 (62.5%)	65 (60.2%)	0.69
History of heart failure^a^	117 (32.7%)	82 (48.0%)	<0.001
Classic cardiovascular risk factors			
Diabetes^b^	98 (27.2%)	51 (30.2%)	0.47
Hypertension^b^	299 (84.2%)	142 (86.6%)	0.48
Hyperlipidemia^b^	237 (67.1%)	112 (70.0%)	0.52
Smoking^b^	76 (22.8%)	28 (18.9%)	0.33
Obesity^c^	145 (41.1%)	75 (47.5%)	0.18
Kidney function			
SCr at admission^d^, mg/dl	0.9 (0.8; 1.0)	1.2 (0.9; 1.3)	<0.001
eGFR at admission, ml/min/1.73m^2^	86.3 (74.9; 96.3)	59.2 (51.3; 80.5)	<0.001
SCr at discharge^e^, mg/dl	0.9 (0.8; 1.0)	1.2 (1.0; 1.4)	<0.001
eGFR at discharge, ml/min/1.73m^2^	85.2 (74.6; 93.8)	57.5 (49.0; 73.8)	<0.001

Data are n (%), median (inter quartile range), analyses restricted to patients without missing values in respective variables

*Abbreviations: CHD* coronary heart disease, *CABG* coronary artery bypass grafting, *PCI* percutaneous coronary intervention, *MI* myocardial infarction, *AMI* acute myocardial infarction, *STEMI* ST-elevation myocardial infarction, *NSTEMI* non-ST-elevation myocardial infarction, *SCr* serum creatinine, *eGFR* estimated glomerular filtration rate

^a^Known at hospital admission or echocardiographic findings of cardiac dysfunction

^b^Known at hospital admission or stated in discharge letter

^c^Body mass index ≥30 kg/m^2^ at hospital admission or stated in discharge letter

^d^First measurement in patient record

^e^Last measurement in patient record

### Physician’s awareness of kidney disease during the (index) hospital stay

Of the 474 patients in whom data on the *kidney module* were available (see Methods), CKD and/or AKI were reported in prominent sections of the hospital discharge letter in 37 (7.8%) of all patients and in 32 (21.5%) of patients with impaired kidney function. While older age, length of hospital stay, diabetes and obesity lost their association in multivariable modeling, worse kidney function at hospital discharge (OR_eGFR_ 0.92 [95% CI 0.89; 0.94]) and more severe episodes of AKI (OR 93 [10; 848]) remained independently related to *physician’s awareness of kidney disease*. Moreover, in patients in whom the index event was the primary diagnosis of CHD, it was less likely (OR 0.38 [0.14; 1.00]) that impaired kidney function was mentioned in the discharge letter (Table 2). In sensitivity analyses, these findings were similar when imputing the missing values (data not shown).

**Table 2:** Determinants of physician’s awareness of kidney disease^a^ at the EUROASPIRE IV index hospital stay (logistic regression)

	Univariable		Multivariable^b^	
	OR (95% CI)	*p*-value	OR (95% CI)	*p*-value
Patient’s age (index) [per year]	1.06 (1.01; 1.11)	<0.01	0.98 (0.92;1.04)	0.56
Patient’s sex [male vs. female]	1.10 (0.44; 2.72)	0.84	–	–
Length of hospital stay [log(d)]	1.58 (1.15; 2.18)	<0.01	1.25 (0.83; 1.89)	0.29
Therapy (max.) [vs. no intervention)]		0.10	–	–
PCI/stent vs. conservative				
CABG vs. conservative	0.49 (0.21; 1.12)			
	1.07 (0.39; 2.96)			
AMI during hospital stay	1.49 (0.76; 2.94)	0.24	–	–
Index hospital stay as primary CHD event [yes vs. no]	0.32 (0.14; 0.75)	<0.01	0.38 (0.14; 1.00)	0.05
History of CHF^c^	1.12 (0.56; 2.21)	0.76	–	–
Cardiovascular risk factors				
Diabetes^d^ (index)	2.57 (1.30; 5.07)	<0.01	1.85 (0.77; 4.44)	0.17
Hypertension^d^ (index)	2.12 (0.63; 7.09)	0.22	–	–
Hyperlipidemia^d^ (index)	2.21 (0.95; 5.17)	0.07	–	–
Smoking (index)^d^	0.35 (0.10; 1.16)	0.08	–	–
Obesity^e^ (index)	2.23 (1.11; 4.48)	0.02	1.34 (0.58; 3.11)	0.49
Kidney function				
eGFR at discharge [per ml/min/1.73m^2^]	0.93 (0.91; 0.95)	<0.001	0.92 (0.89; 0.94)	<0.001
AKI-stages [vs.no AKI]^f^		<0.001		<0.001
Stage 1 [vs. no AKI]	1.51 (0.59; 3.85)		0.38 (0.12; 1.22)	
Stage 2/3 [vs. no AKI]	54.7 (10.8; 277.7)		92.7 (10.1; 847.9)	

Data are odds ratio (OR) with respective 95% confidence interval (CI) and p-value

*Abbreviations: CHD* coronary heart disease, *CABG* coronary artery bypass grafting, *PCI* percutaneous coronary intervention, *eGFR* estimated glomerular filtration rate, *AKI* acute kidney injury, *CKD* chronic kidney disease

^a^CKD and or AKI explicitly mentioned (diagnoses, summary) in the discharge letter of the index hospital stay; analyses based on *n* = 474 patients in whom data on the *kidney module* were available (see Methods)

^b^Multivariable model of backward selection, included are variables with significant (*p* < 0.05) association in univariable analysis; p for exclusion 0.05; non-significant associations displayed italic with OR (95% CI) and *p*-value when variable left the model. Order of exclusion: (1) age; (2) obesity; (3) length of hospital stay; (4) diabetes

^c^Known at hospital admission or echocardiographic findings of cardiac dysfunction

^d^Known at hospital admission or stated in discharge letter

^e^Body mass index ≥30 kg/m^2^ at hospital admission or stated in discharge letter

^f^SCr increase of ≥0.3 mg/dl within 48 h or SCr-increase of 1.5–1.99× baseline SCr within 7 days (stage 1), SCr-increase of 2.0–2.9× baseline SCr (stage 2) and SCr-increase ≥3.0× baseline or SCr >4 mg/dl or dialysis (stage 3)

Of patients admitted to the University Hospital Würzburg (*n* = 498) that had CKD or experienced AKI during the index hospital stay (*n* = 162), relevant ICD codes were applied to 117 (72.2%) patients after discharge. Correct coding was particularly observed in those patients with more severe stages of AKI (100% in stage 2, 75% in stage 3) and CKD (76.9% in stage G3a, 95.2% in G3b, 100% in G4 and 100% in G5).

### Prevalence of chronic kidney disease at the study visit

The study visit was performed on average 1.8 years (1.1; 2.5) after the index hospital stay. At the study visit, 530 of the 536 German patients had SCr values available, with a median of 1.0 (0.9; 1.2) mg/dl reflecting an eGFR of 74.1 (60.0; 85.3) ml/min/1.73 m^2^. ACR was measured in 526 subjects (98.1%), with a median of 4.9 (1.3; 15.8) mg/g. One patient was on chronic hemodialysis treatment (started already prior to the index-hospital stay). According to KDIGO classification of CKD (Fig. 1), 127 (24.4%) individuals had eGFR <60 ml/min/1.73m^2^ and another 48 subjects (9.2%) had largely preserved eGFR but significant albuminuria (i.e. stages G1A3, G2A2, G2A3). Patients with CKD were more likely to be older, with a longer duration of CHD and a history of CABG, heart failure and peripheral artery disease (Table 3). Classic risk factors such as hypertension, diabetes, dyslipidemia and overweight/obesity were also more common in subjects with impaired kidney function, whereas smoking was less prevalent in CKD patients. Primary care regarding CHD was reported to be provided by a cardiologist in 111 (60%) patients with CKD, which on average also had spent a shorter time in education. In those patients, impaired kidney function was reported more frequently in the discharge letter of the index hospital stay. The sensitivity analysis using the eGFR <50 ml/min/1.73m^2^ cut-off identified 65 patients (12.3%). Again, the differences in patient characteristics between of those subjects with more advanced CKD as compared to those with normal kidney function were overall very similar (detailed data not shown) to the results derived from the results presented above (Table 3).

**Table 3:** Patient characteristics at the EUROASPIRE IV study visit by chronic kidney disease^a^

	normal kidney function *n* = 345 (65.1%)	chronic kidney disease *n* = 185 (34.9%)	*p*-value
Age, yrs	65.4 (59.6; 72.2)	73.4 (66.5; 77.0)	<0.001
Male gender	291 (84.4%)	145 (78.4%)	0.09
Total year in education, yrs	12 (11; 15)	11 (11; 14)	<0.001
Education level (higher vs. lower levels^b^)	72 (20.9%)	27 (14.6%)	0.08
History of CHD			
Duration of CHD	2.7 (1.9; 6.4)	3.4 (2.0; 12.9)	<0.01
CHD history			
- CABG	77 (22.3%)	59 (31.9%)	0.02
- PCI/stent	278 (78.6%)	138 (74.6%)	0.12
- AMI	131 (38.0%)	49 (42.7%)	0.30
History of heart failure	40 (11.6%)	38 (20.7%)	<0.01
History of stroke	33 (9.8%)	22 (11.9%)	0.45
History of peripheral artery disease	18 (5.2%)	29 (15.7%)	<0.001
Classic CV risk factors			
BMI [kg/m^2^]	28.0 (25.9; 30.7)	29.5 (27.0; 32.8)	<0.001
Overweight (BMI ≥25)	281 (82.2%)	168 (91.3%)	<0.01
Obesity (BMI ≥30)	105 (30.7%)	86 (46.7%)	<0.001
Blood pressure			
Systolic	133 (122; 148)	138 (128; 152)	<0.01
Diastolic	80 (73; 87)	81 (73; 88)	0.67
Hypertension^c^	142 (41.2%)	96 (52.2%)	0.02
LDL-cholesterol (mmol/l)	2.59 (2.16; 3.14)	2.43 (1.98; 2.98)	0.02
Hyperlipidemia			
LDL cholesterol ≥2.5 mmol/l	188 (56.1%)	77 (45.6%)	0.03
LDL cholesterol ≥1.8 mmol/l	307 (91.6%)	143 (84.6%)	0.02
Diabetes^d^	70 (20.5%)	75 (41.4%)	<0.001
Smoking^e^	49 (14.2%)	14 (7.6%)	0.02
Chronic kidney disease			
SCr_study-visit_ (mg/dl)	1.0 (0.8; 1.0)	1.2 (1.1; 1.4)	<0.001
eGFR_CKD-EPI_ (ml/min/1.73m^2^)	81.0 (71.2; 90.4)	53.9 (46.6; 62.6)	<0.001
ACR_study-visit_ (mg/g)	2.9 (0; 7.4)	20.4 (5.0; 78.5)	<0.001
Information on impaired kidney function in a discharge letter of a hospital stay due to CHD^f^	6 (1.9%)	31 (19.0%)	<0.001

Data are n(%), median (inter quartile range), analyses restricted to patients without missing values in respective variables

*Abbreviations: CHD* coronary heart disease, *CABG* coronary artery bypass grafting, *PCI* percutaneous coronary intervention, *AMI* acute myocardial infarction, *BMI* body mass index, *LDL* low density lipoprotein, *SCr* serum creatinine, *eGFR* estimated glomerular filtration rate, *ACR* urinary albumin/creatinine ratio

^a^CKD (stages CKD-G3 and higher, G2A2, G2A3, G1A3) vs. normal kidney function (G1A1, G1A2, G2A1)

^b^Higher (intermediate between secondary level and university [e.g. technical training], College/University completed, post graduate degree) vs. lower levels of education

^c^As recommended by the German Society of Cardiology as blood pressure ≥ 140/90 mmHg, ≥140/80 mmHg in patients with diabetes, ≥140/85 mmHg in diabetes, ≥150/90 mmHg in patients >80 years, ≥130/90 mmHg in patients with CKD

^d^Self-reported diabetes or impaired fasting glucose/impaired glucose tolerance

^e^Self-reported or CO >10 ppm

^f^EUROASPIRE IV index hospital stay

### Patients’ awareness of chronic kidney disease at the study visit

Data on patient awareness were part of the *kidney module* at the German study center and were thus available in *n* = 474 participants (see Methods). Of those 158 subjects with CKD, 54 (34.2%) patients reported that they had been told about chronic kidney disease, 23 (14.6%) were referred to a renal specialist and 21 (13.3%) had been seen by a specialist. Even in those without overt renal dysfunction at the study visit, 19 (5.5%) reported to be aware of impaired kidney function, 11 (3.2%) were referred and 12 (3.5%) were seen by a specialist. Overall, greater proportions of awareness, referral or specialist care were observed in more advanced stages of kidney disease (p for trend <0.01), however, based on a limited number of observations (Table 4). Accordingly, reduced kidney function (OR_eGFR_ 0.94 [0.92; 0.96]) was also related to an increased level of *patient’s awareness of CKD* in multivariable logistic regression, aside from a history of heart failure (OR 1.99 [1.00; 3.97]), obesity (OR 1.97 [1.07; 3.64]), and the fact that renal impairment was reported in the index discharge letter (OR 5.51 [2.35; 12.9]) (Table 5). Similar results emerged from analyses of the imputed dataset.

**Table 4:** Patient’s awareness of CKD at the EUROASPIRE IV study visit and specialist care by stages of CKD

	CKD G stages at EUROASPIRE IV study visit^a^							
	G1		G2		G3a (*n* = 83)	G3b (*n* = 18)	G4 (*n* = 7)	G5 (*n* = 1)
	All patients (*n* = 84)	Patients with impaired kidney function^b^ (*n* = 1)	All patients (*n* = 275)	Patients with impaired kidney function^b^ (*n* = 47)				
Ever been told by a doctor about impaired kidney function	2 (2.4%)	0	24 (8.7%)	7 (14.9%)	33 (39.8%)	7 (38.9%)	6 (85.7%)	1 (100%)
Recommendation to seek professional advice/referred to kidney specialist^c^	1 (1.2%)	0	12 (4.4%)	2 (4.3%)	11 (13.3%)	5 (27.8%)	4 (57.1%)	1 (100%)
Seen by a kidney specialist^c^	1 (1.2%)	0	13 (4.7%)	2 (4.3%)	9 (10.8%)	4 (22.2%)	5 (71.4%)	1 (100%)

Data are n (% proportions within each category) based on a total of *n* = 474 patients in whom data on the kidney module were available (see Methods). *P*-value for comparison across all categories

*Abbreviations: CKD* chronic kidney disease

^a^CKD G stages according to KDIGO based on eGFR_CKD-EPI_; G1 eGFR ≥90 ml/min/1.73m^2^; G2 60–89, G3a 45–59, G3b 30–44; G4 15–29, G5 < 15 or renal replacement therapy

^b^Definition based on eGFR_CKD-EPI_ and urinary albumin/creatinine (ACR) ratio; KDIGO-stages G1A1, G1A2 and G2A1 considered as normal kidney function, whereas G1A3, G2A2, G2A3 and more severe G-stages are considered as chronic kidney disease (CKD) (see Methods)

^c^Specialist care, e.g. by nephrology, urology

**Table 5:** Determinants of patient’s awareness of CKD^a^ at the EUROASPIRE IV study visit (logistic regression)

	Univariable		Multivariable^b^	
	OR (95% CI)	*P*	OR (95% CI)	*P*
Age, [/yr]	1.05 (1.02; 1.08)	<0.01	*0.97 (0.93; 1.01)*	*0.18*
Male gender [vs. female]	0.59 (0.33; 1.07)	0.08	–	
Education				
Total years [/log(yr)]	0.65 (0.28; 1.50)	0.32	–	
Higher vs. lower levels^c^	0.57 (0.27; 1.20)	0.14	–	
Information on impaired kidney function in a discharge letter of a hospital stay due to CHD^d^	15.8 (7.45; 33.7)	<0.001	5.51 (2.35; 12.9)	<0.001
Primary care for CHD provided by cardiologist [vs. non-cardiologist]	0.88 (0.52; 1.48)	0.62	–	–
History of CHD				
CHD duration [/log(yr)]	1.30 (1.01; 1.66)	0.04	*0.89 (0.64; 1.23)*	*0.47*
CABG ever	1.52 (0.88; 2.63)	0.13	–	–
History of heart failure	2.43 (1.36; 4.34)	<0.01	1.99 (1.00; 3.97)	0.05
History of peripheral artery disease	2.19 (1.07; 4.48)	0.03	*0.83 (0.31; 2.19)*	*0.71*
Diabetes^e^	1.23 (0.71; 2.13)	0.45	–	
Smoking^f^	0.81 (0.37; 1.78)	0.60	–	
Dyslipidemia^g^	1.37 (0.80; 2.34)	0.25	–	
Obesity^h^	1.93 (1.16; 3.21)	0.01	1.97 (1.07; 3.64)	0.03
eGFR_CKD_EPI_ at study visit [/ml/min/1.73m^2^]	0.93 (0.91; 0.95)	<0.001	0.94 (0.92; 0.96)	<0.001
ACR at study visit [/ log(mg/g)]	1.09 (1.01; 1.17)	0.02	*0.99 (0.91; 1.08)*	*0.88*

Data are odds ratio (OR) with respective 95% confidence interval (CI) and p-value

*Abbreviations: CHD* coronary heart disease, *CABG* coronary artery bypass grafting, *PAD* peripheral artery disease, *eGFR* estimated glomerular filtration rate according to CKD-EPI formula, *AKI* acute kidney injury, *CKD* chronic kidney disease

^a^positive response to “Have you ever been told by a doctor/health care provider that your kidney function is impaired, e.g. not as good as it would be expected?”; analyses based on *n* = 474 patients in whom data on the *kidney module* were available (see Methods)

^b^Multivariable model of backward selection, included are variables with significant (*p* < 0.05) association in univariable analysis; p for exclusion 0.05; non-significant associations displayed italic with OR (95% CI) and *p*-value when variable left the model. Order of exclusion: (1) ACR; (2) history of PAD; (3) duration of CHD; (4) age

^c^Higher (intermediate between secondary level and university [e.g. technical training], College/University completed, post graduate degree) vs. lower levels of education

^d^EUROASPIRE IV index hospital stay

^e^Self-reported diabetes or impaired fasting glucose/impaired glucose tolerance

^f^Self-reported or CO >10 ppm

^g^LDL-cholesterol ≥2.5 mmol/L

^h^Body mass index ≥30 kg/m^2^

## Discussion

In our population of German CHD patients enrolled in EUROASPIRE IV, about one third of patients in considerably stable conditions at the study visit had CKD, but only a third of those reported that they had been told about renal impairment. A substantial proportion of patients experienced AKI (18%) during a hospital stay for CHD and/or was discharged with compromised kidney function (18%). Yet, the discharge letter of these patients prominently mentioned chronic or acute kidney disease only in 20%. In contrast, correct ICD coding of CKD or AKI, which is relevant for reimbursement, was more complete but still suboptimal.

### Awareness of CKD among CHD patients with kidney disease

Although CKD is common and associated with worse prognosis, only a small proportion of patients (<5–30%) are aware of their disease in population-based studies [14, 27], CKD cohorts [15, 28] and in CHD patients [21]. Despite public education programs e.g. in the US or the UK, only little improvement in CKD awareness could be observed [17, 29]. Early diagnosis is needed to inform patients about their disease and initiate appropriate treatment [30]. Patient education should be expected to improve adherence to medication and treatment targets [16, 31], but data in CKD are conflicting. Patients on renal replacement therapy (RRT) with adequate knowledge about their disease and treatment targets including dietary restrictions have a lower mortality risk when compared to less educated patients [32]. In contrast, in earlier stages of CKD, achievement of treatment targets for adequate blood pressure control was not significantly associated with the level of patient’s awareness of CKD [17]. Yet, recent data are encouraging that focused education of the primary care physician (PCP) and the patient including his relatives can indeed improve risk factor control and also slow the progression rate of CKD [33]. As directly from the patent’s perspective, it seems intuitive that one feels more confident if he is aware of a certain condition and is informed and educated accordingly about the treatment options. On the other hand, he might also be frightened by the information on the diagnosis and the disease’s prognosis [31], which may aggravate depressive mood, since depression is a common comorbidity in CHD [34] as well as in CKD [35]. Specialist care, e.g. provided by a nephrologist, may help to adequately inform patients about their individual risk for CKD progression [13], and thus help individualizing the therapeutic strategy and treatment targets. It has been shown that (early) referral to nephrology care can slow CKD progression and is associated with reduced mortality risk once RRT is initiated [36, 37].

In our sample of stable CHD patients, we found 24% with CKD (i.e., applying the commonly used cut-off of eGFR <60 ml/min/1.73m^2^), which is nearly 10-times higher than in recent numbers of the German general population [38]. Another 10% of our sample had albuminuria with preserved eGFR representing those at risk for CKD progression [24, 39]. Of those with CKD, only about a third were aware of their disease and only a minority was being seen by nephrologists, however, with higher likelihood of CKD awareness and specialist care in more severe stages of CKD. The latter observation, however, is based on very few data in the respective categories and need to be interpreted with caution. We could not find significant associations with CKD awareness with the level of education, gender, or diabetic status. Yet, a history of heart failure was related to a higher level of CKD awareness, independently of the severity of CKD. This might be explained by frequent appointments specifically for heart failure and potentially also for cardio-renal syndrome [40] at the cardiologist and the PCP, with an increased likelihood of impaired kidney function being detected, mentioned and discussed during such appointments. Furthermore, we found that patients were more likely to know about impaired kidney function if either CKD or AKI was mentioned in a recent discharge letter. Both factors underline the relationship of patient information as being directly dependent on the physician’s awareness, information and education [41].

### Physician’s awareness of kidney disease during a hospital-stay for CHD

Patients with kidney disease are also at a higher risk for complications in hospital stays for CHD and impaired prognosis after discharge [42, 43]. Causes for affected kidney function are multifactorial, including acutely reduced renal perfusion in myocardial infarction, nephrotoxic contrast application during catheter interventions, but also in elective CABG surgery [25]. These AKI episodes, which might be only temporary changes in kidney function of milder degree (e.g., a rise in SCr by 0.3 mg/dl in AKI stage 1 [25]) are associated with an increased risk for cardiovascular events, cardiac dysfunction, heart failure progression, and risk of hospitalization and death [44].

In general, markers of kidney function at hospital admission and during the early phase of a hospital stay are more likely to be influenced by AKI, whereas kidney function might be improved and stabilized at hospital discharge. While in the core protocol of EUROASPIRE IV only SCr values at hospital admission were collected, at the German study center, we used all SCr measurements during the index hospital stay, thus enabling detailed analysis of the course of kidney function including AKI episodes. Of note, even if all methods were considered (e.g. urinary dip-stick, 24 h urine collection, ACR or total protein/creatinine ratio), in more than half of the patients no measure of proteinuria was available. Therefore, estimating the entire spectrum of kidney disease including proteinuria was impossible.

We found that during the index hospital stay, about 18% of patients experienced AKI, while most episodes were of stage 1, but more severe stages including those needing hemodialysis were observed. Only a very small number of patients had a rise of SCr of exactly 0.3 mg/dl that did not further increase in subsequent laboratory measurements. Also about 18% were found for impaired kidney function at hospital admission and at hospital discharge, respectively. Yet, one third of all patients was detected as having any impairment of kidney function either acute and/or chronic, respectively.

Since the introduction of equations based on SCr to estimating GFR, eGFR is increasingly displayed on routine laboratory reports with every SCr measurement [45], including the recruiting German EUROASPIRE IV centers. Therefore, information on kidney function is routinely visible to the treating physician. Moreover, the role of CKD and AKI as an important comorbid condition is widely discussed in the medical literature. The rationale for using mentioning of CKD or AKI in prominent parts of the hospital discharge letter as a proxy for *physician’s awareness of impaired kidney function* was as follows: First, even slight changes in SCr need to be recognized by the physician as AKI, an important acute complication during the hospital stay. Second, either CKD or AKI reflect important risk factors for both CHD and CKD progression. Since the discharge letter represents the most important document of information transfer from the hospital to the ambulatory setting, the treating physician needs to judge kidney dysfunction as important enough to be clearly reported the discharge letter. In clinical routine in Germany, particularly the first part (diagnoses) and the end of the document (summary and medication) are predominantly being read by PCPs due to time constraints.

We found that the discharge letter reported only one fifth of patients with impaired kidney function (acute or chronic). While comorbid conditions, CHD history or the procedure itself were unrelated to *physician’s awareness*, it was reassuring that higher stages of CKD or AKI increased chances for kidney dysfunction being reported in the discharge letter. Importantly, in patients in whom the index event was the primary diagnosis of CHD, impaired renal function was frequently not reported in the discharge letter. In particular in these patients, comprehensive description of traditional and non-traditional CV risk factors is needed for establishing an individualized treatment concept for optimal secondary CHD prevention [46]. The discharge letter not only addresses the PCP, but also constitutes an important source for information to the patient himself, supporting self-empowerment, self-management and self-monitoring. Since reporting CKD or AKI in the discharge letter relates to patients’ awareness of CKD, raising the *physician’s awareness of kidney disease* may ultimately lead to better informed patients.

### Completeness of ICD-coding for kidney disease in patients with CHD

In 2003/2004, the German Diagnosis Related Group (G-DRG) system replaced the cost-based reimbursement of hospital stays employing ICD diagnoses, procedures, and comorbidities [11]. For each case, coding usually gets completed a few days after discharge, commonly with the help of expert coding assistants. As AKI and CKD increase the amount of reimbursement, adequate coding is highly relevant for the hospital. In our study, CKD and AKI were correctly coded for the majority of patients, in particular in those with advanced stages. However, there were still patients in whom adequate coding of renal function would have increased monetary benefits for the hospital.

### Strengths and limitations

The unique setting of the EUROASPIRE IV study including the *kidney module* at the German study center allows a comprehensive view on CKD awareness among CHD patients and their treating physicians, however, limitations need to be mentioned. First, the study-sample cannot be claimed as representative neither for CHD patients in Germany nor for those admitted for CHD at the recruiting hospitals due to the selection process of centers and the recruitment success [20]. Second, CKD at the EUROASPIRE IV study visit was classified based on a single measurement of SCr and ACR, while usually two independent measurements for adequate assessment of CKD are desired [24]. However, study participants were in apparently stable condition at the time of recruitment. In contrast, as also discussed above, during the index hospital stay, kidney function at admission as well as at discharge might be influenced by acute clinical circumstances, e.g. acute kidney injury at admission or the (prolonged) convalescence of kidney function after AKI. Third, any SCr-based estimation of GFR has limitations, and mild to moderately impaired kidney function may be better described by equations based on Cystatin C [47], which unfortunately was not available. However, we chose the SCr-based CKD-EPI formula as it outperforms the MDRD formula in particular in GFR between 60 and 90 ml/min/1.73m^2^ [48]. Fourth, the patient’s knowledge of having been told about CKD may by influenced by multiple factors, including the setting of a study visit, therefore recall bias may apply. In addition, particularly in older subjects, a slight deterioration of kidney function might be considered as normal, age-related decline in GFR. Fifth, the number of variables tested in logistic regression analyses may be considered as too high, thus finding and missing associations by chance and limited power is surely possible. Finally, the substantial discrepancy between correct ICD-coding and presence of impaired kidney function in the discharge letter might be explained the fact that the discharge letter is predominantly prepared by junior physicians and may not reflect awareness by the more senior physician involved in care decisions, whereas the coding is supported by professional coders who may detect comorbid conditions that may have not been in the primary focus of the patient’s therapeutic care.

## Conclusion

In patients with CHD, mild to moderate CKD is a common comorbidity, but only few patients are aware of their renal dysfunction. Furthermore, in only a limited number of patients, renal impairment is being reported in hospital discharge letters, whereas the majority of subjects appears correctly ICD-coded. Stringent reporting of CKD and AKI may improve information transfer to care givers in the outpatient setting. How this may further lead to better informed patients, higher attainment of treatment targets, and improved management of both CKD and CHD should be focus of future studies.

## Abbreviations

ACR: Albumin / creatinine ratio
AKI: Acute kidney injury
AMI: Acute myocardial infarction
BMI: Body mass index
CABG: Coronary artery bypass grafting
CHD: Coronary heart disease
CHF: Congestive heart failure
CI: Confidence interval
CKD: Chronic kidney disease
CKD-EPI: Chronic kidney disease epidemiology collaboration
CVD: Cardiovascular disease
eGFR: Estimated glomerular filtration rate
ESC: European Society of Cardiology
HDL-C: High density lipoprotein cholesterol
IQR: Inter-quartile range
KDIGO: Kidney disease – improving global outcomes
LDL-C: low density lipoprotein cholesterol
OR: Odds ratio
PCI: Percutaneous coronary intervention
SCr: Serum creatinine
SD: Standard deviation
